# Patient healthcare experiences of cancer hospitals in China: A multilevel modeling analysis based on a national survey

**DOI:** 10.3389/fpubh.2023.1059878

**Published:** 2023-02-22

**Authors:** Meicen Liu, Linlin Hu, Yue Xu, Yue Wang, Yuanli Liu

**Affiliations:** School of Health Policy and Management, Chinese Academy of Medical Sciences and Peking Union Medical College, Beijing, China

**Keywords:** patient experience, cancer, tertiary hospital, hospital performance, China

## Abstract

**Importance:**

Patient satisfaction is a crucial indicator for assessing quality of care in healthcare settings. However, patient satisfaction benchmark for cancer hospitals in China is not established.

**Objective:**

To examine patient satisfaction levels in tertiary cancer hospitals in China, and inter-hospital variations after case-mix adjustment.

**Design:**

A nationwide cross-sectional hospital performance survey conducted from January to March 2021.

**Settings:**

At 30 tertiary cancer hospitals in China.

**Participants:**

A total of 4,847 adult inpatients consecutively recruited at 30 tertiary cancer hospitals were included.

**Exposures:**

Patient characteristics included demographic characteristics (sex, age, education, and annual family income), clinical characteristics (cancer type, cancer stage, self-reported health status, and length of stay), and actual respondents of questionnaire.

**Main outcomes and measures:**

Patient satisfaction was measured using 23 items covering five aspects, administrative process, hospital environment, medical care, symptom management, and overall satisfaction. Responses to each item were recorded using a 5-point Likert scale. Patient satisfaction level for each aspect was described at individual and hospital levels. Using multilevel logistic regression, patient characteristics associated with patient satisfaction were examined as case-mix adjusters and inter-hospital variation were determined.

**Results:**

The satisfaction rates for symptom management, administrative process, hospital environment, overall satisfaction, and medical care aspects were 74.56, 81.70, 84.18, 84.26, and 90.86% with a cut-off value of 4, respectively. Significant predictors of patient satisfaction included sex, age, cancer type, cancer stage, self-reported health status, and actual respondent (representative or patient) (all *P* < 0.05). The ranking of the hospitals' performance in satisfaction was altered after the case-mix adjustment was made. But even after the adjustment, significant variation in satisfaction among hospitals remained.

**Conclusions and relevance:**

This study pointed to symptom management as a special area, to which a keen attention should be paid by policymakers and hospital administrators. Significant variation in satisfaction among hospitals remained, implying that future studies should examine major factors affecting the variation. In review, target interventions are needed in low-performing hospitals.

## Key points

Question: What is the patient satisfaction level with different healthcare aspects in China's cancer hospitals? Does the patient satisfaction vary across hospitals?

Findings: Drawing from a national hospital performance survey that included 4,847 inpatients in China's 30 cancer hospitals, patient satisfaction level with clinical aspects of healthcare is found to be high, while there was a perceived need for improvement in symptom management and the cumbersome hospital admission process. The ranking of the hospitals' performance in satisfaction was altered after the case-mix adjustment was made, while significant variation in satisfaction among hospitals remained.

Meanings: This study pointed to symptom management as a special area, to which a keen attention should be paid by policymakers and hospital administrators and discrepancy of hospital performance in patient satisfaction remains to be narrowed.

## Introduction

Cancer is the second leading cause of death and a major contributor to the disease burden worldwide and in China (1). Cancer patients take efforts in seeking hospitals offering safe, effective and comfortable care. In China, cancer patients could receive treatment and care in various hospitals, among which cancer hospitals provide the most specialized and skilled oncology treatment and deliver a large proportion of cancer care. Besides, cancer hospitals in China were graded as three levels based on their technology and resources, including primary, secondary and tertiary cancer hospitals (2). The tertiary cancer hospitals represent China's national and regional medical centers for cancer care and regularly offer treatments for numerous patients with more severe and complicated conditions. In China with the increase of cancer patients, annual admissions of patients by cancer hospitals have increased from 1.10 million to 3.24 million from 2010 to 2020, which posed a great challenge for these hospitals (3).

Patients' perception of their care is a vital source of healthcare quality assessment alone or in combination with clinical outcomes (4–6). Patient experience or satisfaction as the most commonly used indicator of patients' perception has become a key component of performance assessment and certification in healthcare settings (7, 8), helping guide quality improvement actions, regulations, and incentive payments (9, 10). National initiatives involving patient experience surveys have been developed for decades, such as the Hospital Consumer Assessment of Healthcare Providers and Systems (HCAHPS) survey in the USA, the National Health Service (NHS) National Inpatient Survey in England, and other surveys in other countries or regions (11–13). Several initiatives in cancer care settings also have been gradually developed, including the CAHPS (Consumer Assessment of Healthcare Providers and Systems) Cancer Care Survey, NCPES (National Cancer Patient Experience Survey), and ECCQI (European Cancer Consumer Quality Index) (14–19). In China some exploratory studies were conducted on this topic but usually with small sample and either in one single cancer center or on single cancer type (20–23). The nationwide, multi-center surveys covering patients with various cancer types in China are lacking.

Previous studies on cancer patient experience mainly focused on impact factors and subgroup differences in patient satisfaction (24, 25). Age, sex, education, income, race or ethnicity, disease characteristics, treatment files, and survey methods are associated with satisfaction in some advanced countries and regions (24–28). These studies mainly focused on the level of patient satisfaction and associated factors, but did not explored the inter-hospital variation, which is important for targeted improvement of cancer care for specific hospital. Case-mix adjustment has been widely used in hospital comparison studies and it helps precisely measure inter-hospital variation after removing the effect of patient constitution (29–34).

Based on a multi-center cross-sectional patient survey in China, this study measured cancer patient satisfaction in tertiary cancer hospitals across China. We employed a case-mix model for each aspect of patient satisfaction, demonstrating the effects of the model on hospital comparisons. Inter-hospital variation in patient satisfaction after case-mix adjustment was determined.

## Methods

Based on the national hospital performance survey, a multi-center cross-sectional study of patient satisfaction in tertiary cancer hospitals was conducted between January and March 2021 (13, 35, 36). This survey was commissioned by the China National Health Commission to investigate patients' perceptions and the performance of tertiary public hospitals. This study followed the Reporting of Observational Studies in Epidemiology (STROBE) reporting guideline.

### Sampling

A total of 30 tertiary cancer hospitals across 28 provinces in China were included, covering all national and most provincial-level cancer hospitals, accounting for approximately half of tertiary cancer hospitals in China. The sample size was at least 1,746 to estimate a satisfaction rate of 80% with a precision of plus or minus 2% at the 95% confidence level; and set an invalid proportion of collected data of 10%. We set a minimum sample size of 150 inpatients in each of the 30 hospitals, so the planned sample size in 30 tertiary cancer hospitals was 4,500 in total.

### Data collection

At least 150 inpatients per hospital were consecutively recruited from January to March 2021. Patients on the day of or the day prior to discharge, aged 18 and over and with clear consciousness were invited to participate in this survey. They were asked to fill in the questionnaire using their electronic devices under the guide of trained investigators. If patients could not respond to the questionnaire independently due to inconvenience or inability, the family members present at the hospital provided responses based on the patients' perspectives. All patients provided informed consent for their participation in the study. Finally, a total of 4,847 eligible adult patients took part in this survey and were included in our analyses.

### Survey instrument and variables

The patient satisfaction questionnaire regarding cancer care was designed based on international surveys [including the CAHPS (37, 38), NCPES (16, 28), ECCQI (19), and other area-related surveys (11, 12, 39, 40)] and domestic policies related to improving care quality (13, 18). Cancer patient satisfaction in this questionnaire were measured with 23 items over five aspects including administrative process, hospital environment, medical care, symptom management and overall satisfaction. Responses to each item were recorded using a 5-point Likert scale (5-strongly satisfied to 1-strongly dissatisfied) or “not applicable.” Table 1 showed the items and average score of each aspect. Every aspect got its score ranged from 1 to 5 based on the average score of corresponding satisfaction items. Higher scores represent higher patient satisfaction.

**Table 1:** Patient satisfaction, internal consistency, and average aspect scores.

**Aspect**	**Items**	**Questions**	**Cronbach's α**	**Aspect score mean, SD**
Administrative process	4	-I am satisfied with the waiting time for admission	0.794	4.67 (0.52)
		-I am satisfied with the admission procedure		
		-I am satisfied with the accessibility of medical complains		
		-I am satisfied with the punctuality of surgery		
Hospital environment	5	-I am satisfied with the cleanness and absence of smells in ward and toilet	0.880	4.71 (0.46)
		-I am satisfied with the quietness of the ward		
		-I am satisfied with the fall prevention equipment in the hospital		
		-I am satisfied with the signage in the hospital		
		-I am satisfied with the accessibility of hand-washing solution in the ward		
Medical care	9	-I am satisfied that the doctor took my treatment preferences into account	0.946	4.82 (0.36)
		-I am satisfied with the protection of patient's privacy of medical staffs		
		-I am satisfied with medication instructions of medical staffs		
		-I am satisfied that the doctor carefully explain about treatment schemes (pros and cons)		
		-I am satisfied with the doctors' care		
		-I am satisfied with the doctors' careful inquiry about my illness conditions		
		-I am satisfied with the nurses' timely help when I need		
		-I am satisfied with the courtesy and respect of medical staffs		
		-I am satisfied with the nurses' manners		
Symptom management^a^	3	-I am satisfied with psychological counseling regarding stress, anxiety and depression	0.942	4.67 (0.61)
		-I am satisfied with diet instructions regarding nausea and vomiting		
		-I am satisfied with pain management regarding severe hurt		
Overall satisfaction	2	-Overall, I am satisfied with this hospitalization	0.753	4.77 (0.43)
		-I would like to recommend the hospital to my relatives and friends		

a Sample size of symptom management was 2,276 because not all patients had undergone clinical symptoms surveyed.

This questionnaire was validated through three rounds of expert consultation and a small-scale pilot test. The internal consistency of five aspects was tested using Cronbach's α coefficient. Cronbach's α values were 0.753–0.942 at an acceptable level, which indicating a sound reliability of the survey questionnaire.

This questionnaire also collected patient information including demographic characteristics (sex, age, education, and annual family income), clinical characteristics (cancer type, cancer stage, self-reported health status, and length of stay), and actual respondents (whether the patient or a representative completed the questionnaire). Actual respondents were categorized as patients or representatives. Representatives completed the questionnaire when patients were unavailable or unable to respond independently.

### Statistical analysis

We transferred the patient satisfaction score for each aspect into a binary variable at a cutoff point of 4 (patient satisfaction = 1 if average score >4). The patient satisfaction rates for each aspect at individual level were calculated and those at hospital level were depicted at box graphs. The Pearson's chi-square tests were used to compare the satisfaction rate for each aspect and preliminarily identified the relationship between patient characteristics and satisfaction level.

Because our data have a hierarchical structure and patients were nested in multiple hospitals, multilevel regressions were used to explore case-mix factors related to patient satisfaction. We performed multilevel logistic regression for each aspect. All covariates identified in this paper were included in multilevel logistic regression analyses for all these factors were statistically significant or clinical significance. Intraclass correlation coefficient (ICC) was inspected, which indicated the extent to which the overall variation in patient satisfaction could be attributed to hospital effect (41). The ICCs ranged from 6.9 to 15.5% across five aspects evaluated in this study, reconfirming the necessity for multilevel modeling (Table 3).

The 30 tertiary hospitals included 3 national-level hospitals, 27 provincial-level hospitals. The 3 national-level hospitals were labeled “NA,” “NB,” and “NC,” and the other cancer hospitals were labeled “A,” “B,” “C,” etc. A caterpillar plot for each aspect was generated to depict inter-hospital variation in patient satisfaction (41). The y-axis shows the estimate of the hospital residuals with 95% confidence intervals for patient satisfaction after modeling multilevel multivariate regressions; the x-axis shows the hospital rank based on the hospital's residual, ranging from low to high. The residuals represent hospital departures from the overall mean, so a hospital whose confidence interval does not overlap the line at zero (zero line representing the mean satisfaction rate across sampled hospitals) is said to differ significantly from the average at 5% level. At the left-hand side of the plot, there is a cluster of hospitals whose mean satisfaction rate is lower than average (categorized as worse); at the other extreme, there is a cluster with above-average satisfaction rate (categorized as better); when overlapping the line at zero, there is a cluster with average satisfaction rate (categorized as average) (42).

Statistical analyses were conducted using Stata/SE 15.0 software (Stata Corp. LP, College Station, TX, USA). All tests were two-sided, and *P* < 0.05 indicated statistical significance.

## Results

### Patient characteristics

Of the 4,847 patients sampled in 30 hospitals, 2,291 (47.27%) were male, 2,637 (54.40%) were 45–64 years of age, and 2,631 (54.28%) had a junior or high school education. Lung, digestive tract, or breast/cervical cancer was present in 14.98, 19.89, and 17.80% of the patients, respectively; 30.62% had stage 3–4 cancer, and the hospital stay exceeded 14 days in 20.51% (Table 2). Some patients did not experience clinical symptoms during their hospital stay; thus, the symptom management aspect did not apply to them. The symptom management aspect analysis included 2,276 patients.

**Table 2:** Patient characteristics and the rate of patient satisfaction.

**Specification**	**Number of inpatients, *N* (%)**	**Administrative process**	**Hospital environment**	**Medical care**	**Symptom management^a^**	**Overall satisfaction**
Total	4,847 (100)	3,960 (81.70)	4,080 (84.18)	4,404 (90.86)	1,697 (74.56)	4,084 (84.26)
**Sex**						
Male	2,291 (47.27)	1,841 (80.36)	1,925 (84.02)	2,072 (90.44)	808 (75.30)	1,923 (83.94)
Female	2,556 (52.73)	2,119 (82.90)	2,155 (84.31)	2,332 (91.24)	889 (73.90)	2,161 (84.55)
*P*		**0.02**	0.79	0.34	0.44	0.56
**Age**						
18–44	1,190 (24.55)	968 (81.34)	983 (82.61)	1,077 (90.50)	394 (73.78)	1,001 (84.12)
45–64	2,637 (54.40)	2,173 (82.40)	2,263 (85.82)	2,417 (91.66)	946 (75.20)	2,239 (84.91)
65–85	1,020 (21.04)	819 (80.29)	834 (81.76)	910 (89.22)	357 (73.76)	844 (82.75)
*P*		0.31	**0.002**	0.06	0.63	0.27
**Education**						
College or above	1,280 (26.41)	1,064 (83.13)	1,090 (85.16)	1,175 (91.80)	430 (74.14)	1,102 (86.09)
Junior or high school	2,631 (54.28)	2,153 (81.83)	2,229 (84.72)	2,399 (91.18)	939 (75.30)	2,217 (84.26)
Primary School or less	936 (19.31)	743 (79.38)	761 (81.30)	830 (88.68)	328 (73.05)	765 (81.73)
*P*		0.08	**0.03**	**0.03**	0.62	**0.02**
**Annual family income**						
<30,000	1,359 (28.04)	1,084 (79.76)	1,118 (82.27)	1,214 (89.33)	508 (73.41)	1,108 (81.53)
30,000–60,000	1,626 (33.55)	1,298 (79.83)	1,348 (82.90)	1,472 (90.53)	563 (73.69)	1,364 (83.89)
≥60,000	1,862 (38.42)	1,578 (84.74)	1,614 (86.68)	1,718 (92.27)	626 (76.34)	1,612 (86.57)
*P*		**<0.001**	**0.001**	**<0.001**	0.34	**<0.001**
**Cancer type**						
Lung cancer	726 (14.98)	565 (77.82)	602 (82.92)	658 (90.63)	292 (73.55)	605 (83.33)
Digestive tract cancer (esophagus, gastric, colorectal)	964 (19.89)	795 (82.47)	815 (84.54)	878 (91.08)	394 (73.64)	819 (84.96)
Breast or cervical cancer	863 (17.80)	683 (79.14)	717 (83.08)	768 (88.99)	288 (68.90)	712 (82.50)
Other types cancer	1,528 (31.52)	1,291 (84.49)	1,302 (85.21)	1,420 (92.93)	585 (78.84)	1,323 (86.58)
Non-cancer	766 (15.80)	626 (81.72)	644 (84.07)	680 (88.77)	138 (75.00)	625 (81.59)
*P*		**0.001**	0.56	**0.004**	**0.005**	**0.01**
**Cancer stage**						
0–2	1,403 (28.95)	1,167 (83.18)	1,198 (85.39)	1,283 (91.45)	518 (76.97)	1,206 (85.96)
3–4	1,484 (30.62)	1,227 (82.68)	1,277 (86.05)	1,384 (93.26)	653 (76.11)	1,296 (87.33)
Unknown	1,960 (40.44)	1,566 (79.90)	1,605 (81.89)	1,737 (88.62)	526 (70.60)	1,582 (80.71)
*P*		**0.03**	**0.001**	**<0.001**	**0.01**	**<0.001**
**Self-reported health status**						
Worse	17,84 (36.81)	1,439 (80.66)	1,462 (81.95)	1,611 (90.30)	695 (72.32)	1,473 (82.57)
Moderate	1,684 (34.74)	1,372 (81.47)	1,410 (83.73)	1,517 (90.08)	576 (73.94)	1,399 (83.08)
Better	1,379 (28.45)	1,149 (83.32)	1,208 (87.60)	1,276 (92.53)	426 (79.48)	1,212 (87.89)
*P*		0.15	**<0.001**	**0.04**	**0.009**	**<0.001**
**Length of stay**						
1–14 days	3,853 (79.49)	3,187 (82.71)	3,279 (85.10)	3,531 (91.64)	1,322 (75.93)	3,270 (84.87)
>14 days	994 (20.51)	773 (77.77)	801 (80.58)	873 (87.83)	375 (70.09)	814 (81.89)
*P*		**<0.001**	**0.001**	**<0.001**	**0.007**	**0.02**
**Respondent**						
Representative	1,470 (30.33)	1,161 (78.98)	1,195 (81.29)	1,330 (90.49)	543 (70.70)	1,212 (82.45)
Patient	3,377 (69.67)	2,799 (82.88)	2,885 (85.43)	3,074 (91.03)	1,154 (76.53)	2,872 (85.05)
*P*		**0.001**	**<0.001**	0.54	**0.003**	**0.02**

Bold P-values < 0.05.

a Sample size of symptom management was 2,276 because not all patients had undergone clinical symptoms surveyed.

### Patient satisfaction level

The satisfaction scores associated with the administrative process, hospital environment, medical care, symptom management, and overall satisfaction were 4.67, 4.71, 4.82, 4.67, and 4.77, respectively (Table 1). The respective satisfaction rates were 81.70, 84.18, 90.86, 74.56, and 84.26% (Table 2). The satisfaction level was highest in the medical care aspect and comparatively lower in the symptom management and administrative process aspects for respondents. Besides, patients in different sociodemographic groups showed different satisfaction levels. For example, female patients expressed higher satisfaction in administrative process aspect, and patients in the middle age expressed higher satisfaction in hospital environment aspect.

**Figure 1:**
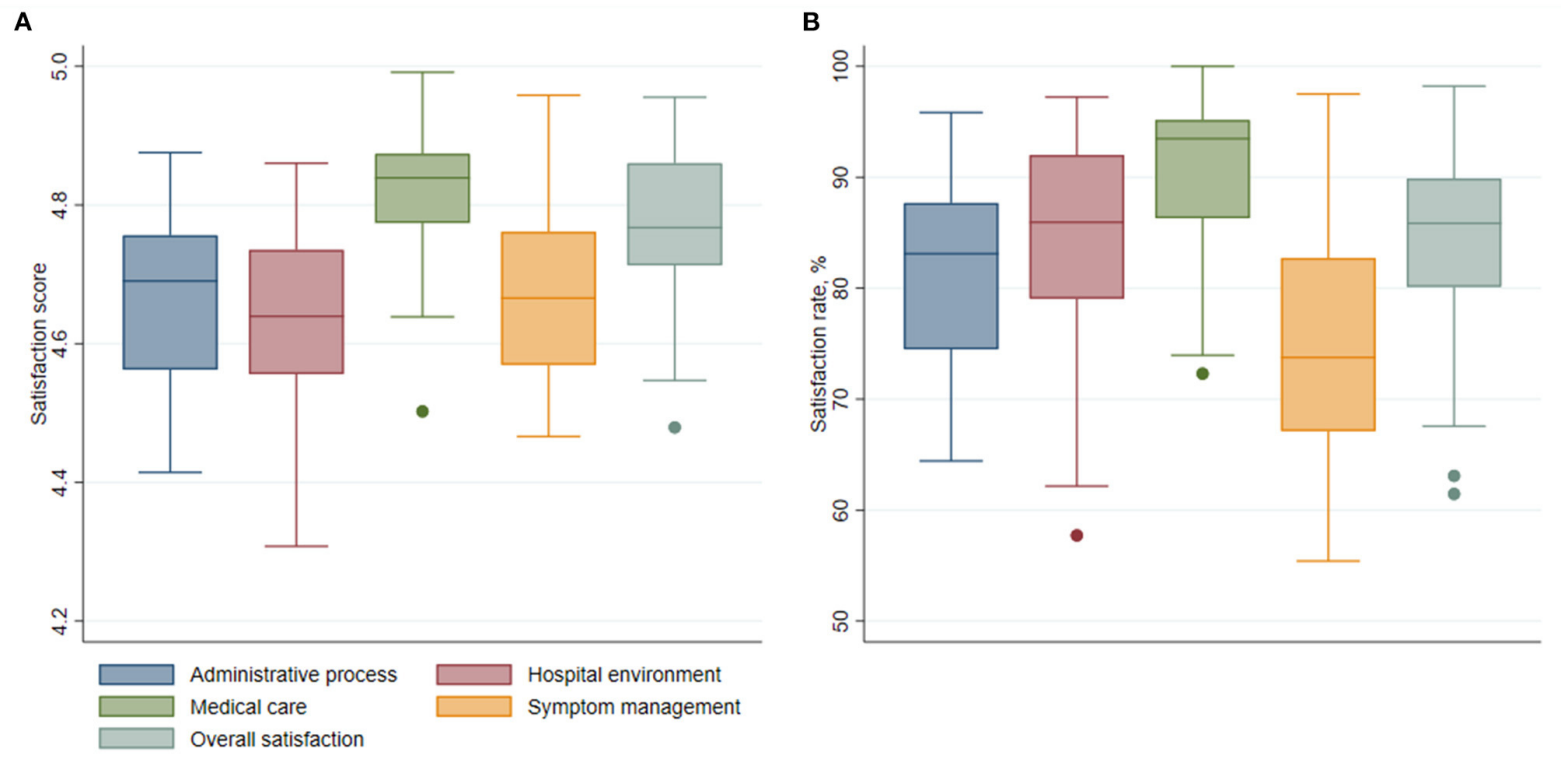
Patient satisfaction aggregated at hospital level over five aspects. **(A)** Box plot of patient satisfaction scores over five aspects. **(B)** Box plot of patient satisfaction rates over five aspects.

The aggregated satisfaction scores and rates for each hospital are shown in Figure 1. At the hospital level, the symptom management and process aspects also showed the lower level of satisfaction, and the medical care aspect showed the highest. The aggregated satisfaction scores ranged from 4.31 to 4.99 in all aspects across hospitals; and the satisfaction rates ranged from 55.41 to 100.0%, indicating substantial differences in hospital performance (Supplementary Table 1).

### Predictors on patient satisfaction

In the multivariate multi-level regression models, statistically significant predictors for at least one aspect were sex, age, cancer type, cancer stage, self-reported health status, and actual respondent (Table 3). Patients aged 45–64 years were 1.40 times more likely to be satisfied with the hospital environment than those aged <45 years (*OR*: 1.40, 95% *CI*: 1.14–1.73). Compared with those with worse self-reported health status, patients with better self-reported health status were 1.55 (95% *CI*: 1.25–1.92) and 1.52 (95% *CI*: 1.16–1.99) times more likely to be satisfied with the hospital environment and symptom management, respectively, and their overall satisfaction was 1.60 (95% *CI*: 1.29–1.99) times higher.

**Table 3:** Multilevel logistic regression of patient satisfaction (*OR* with 95% confidence interval).

**Specification**		**Administrative process**	**Hospital environment**	**Medical care**	**Symptom management^a^**	**Overall satisfaction**
Sex	Female	1 [Reference]	1 [Reference]	1 [Reference]	1 [Reference]	1 [Reference]
	Male	**0.73** (0.61, 0.87)^***^	0.90 (0.75, 1.09)	**0.76** (0.60, 0.96)^*^	0.93 (0.74, 1.16)	0.88 (0.73, 1.06)
Age	18–45	1 [Reference]	1 [Reference]	1 [Reference]	1 [Reference]	1 [Reference]
	45–64	1.19 (0.98, 1.45)	**1.40** (1.14, 1.73)^***^	1.19 (0.91, 1.55)	1.13 (0.87, 1.45)	1.13 (0.92, 1.39)
	65–85	1.09 (0.85, 1.39)	1.11 (0.86, 1.45)	0.91 (0.66, 1.27)	1.10 (0.79, 1.52)	1.01 (0.78, 1.32)
Education	College or above	1 [Reference]	1 [Reference]	1 [Reference]	1 [Reference]	1 [Reference]
	Junior or high school	1.02 (0.84, 1.25)	1.04 (0.84, 1.29)	0.96 (0.74, 1.27)	1.12 (0.87, 1.44)	0.92 (0.74, 1.14)
	Primary School or less	0.91 (0.70, 1.19)	0.89 (0.67, 1.18)	0.72 (0.51, 1.02)	1.06 (0.76, 1.49)	0.80 (0.60, 1.06)
Monthly family income	<30,000	1 [Reference]	1 [Reference]	1 [Reference]	1 [Reference]	1 [Reference]
	30,000–60,000	0.92 (0.76, 1.11)	0.92 (0.75, 1.13)	1.06 (0.82, 1.36)	0.94 (0.73, 1.20)	1.03 (0.84, 1.26)
	≥60,000	1.15 (0.93, 1.43)	1.08 (0.86, 1.36)	1.12 (0.84, 1.49)	1.04 (0.79, 1.37)	1.11 (0.88, 1.39)
Cancer type	Lung cancer	**0.63** (0.50, 0.80)^***^	0.82 (0.63, 1.06)	0.75 (0.54, 1.06)	**0.73** (0.54, 0.98)^*^	**0.75** (0.58, 0.98)^*^
	Digestive tract cancer (esophagus, gastric, colorectal)	0.89 (0.71, 1.11)	0.96 (0.76, 1.22)	0.84 (0.61, 1.15)	0.77 (0.58, 1.02)	0.91 (0.72, 1.16)
	Breast or cervical cancer	**0.56** (0.44, 0.72)^***^	0.78 (0.60, 1.02)	**0.49** (0.35, 0.68)^***^	**0.54** (0.40, 0.74)^***^	**0.64** (0.49, 0.83)^***^
	Other types	1 [Reference]	1 [Reference]	1 [Reference]	1 [Reference]	1 [Reference]
	Non-cancer	1.07 (0.80, 1.43)	1.21 (0.89, 1.64)	1.01 (0.69, 1.47)	1.17 (0.76, 1.79)	1.22 (0.90, 1.65)
Cancer stage	0–2	1 [Reference]	1 [Reference]	1 [Reference]	1 [Reference]	1 [Reference]
	3–4	0.92 (0.75, 1, 14)	1.10 (0.88, 1.38)	1.28 (0.95, 1.71)	0.93 (0.72, 1.19)	1.13 (0.90, 1.42)
	unknown	**0.81** (0.65, 0.99)^*^	**0.79** (0.63, 0.99)^*^	0.80 (0.61, 1.06)	**0.66** (0.51, 0.87)^**^	**0.70** (0.56, 0.87)^***^
Self-reported health status	Worse	1 [Reference]	1 [Reference]	1 [Reference]	1 [Reference]	1 [Reference]
	Moderate	1.03 (0.86, 1.24)	1.15 (0.95, 1.39)	0.99 (0.78, 1.26)	1.09 (0.87, 1.36)	1.06 (0.88, 1.28)
	Better	1.14 (0.94, 1.39)	**1.55** (1.25, 1.92)^***^	1.25 (0.95, 1.64)	**1.52** (1.16, 1.99)^**^	**1.60** (1.29, 1.99)^***^
Length of stay	1–14 days	1 [Reference]	1 [Reference]	1 [Reference]	1 [Reference]	1 [Reference]
	>14 days	0.86 (0.72, 1.03)	0.91 (0.75, 1.11)	0.85 (0.67, 1.08)	0.83 (0.66, 1.04)	0.99 (0.81, 1.21)
Respondent	Patient	1 [Reference]	1 [Reference]	1 [Reference]	1 [Reference]	1 [Reference]
	Representative	**0.82** (0.69, 0.98)^*^	**0.80** (0.66, 0.97)^*^	1.07 (0.84, 1.36)	**0.76** (0.61, 0.95)^*^	0.88 (0.73, 1.07)
ICC(%)^b^		0.108 (0.063, 0.181)	0.155 (0.093, 0.247)	0.161 (0.092, 0.265)	0.069 (0.034, 0.136)	0.144 (0.084, 0.235)

a Sample size of symptom management was 2,276 because not all patients had undergone clinical symptoms surveyed.

* P < 0.05,

** P < 0.01,

*** P < 0.001.

b Represent ICC and its 95%confidence interval after case-mix adjustment. Bold OR with 95% confidence interval is beyond or below 1.

ICC, intraclass correlation coefficient.

Male patients had 27% (*OR*: 0.73, 95% *CI*: 0.61–0.87) and 24% (*OR*: 0.76, 95% *CI*: 0.60–0.96) lower odds of being satisfied with the administrative process and medical care than their counterparts. Compared with those with other cancer types, patients with lung cancer had 37% (*OR*: 0.63, 95% *CI*: 0.50–0.80) and 27% (*OR*: 0.73, 95% *CI*: 0.54–0.98) lower odds of being satisfied with the administrative process and symptom management, respectively, and their overall satisfaction was 25% (*OR*: 0.75, 95% *CI*: 0.58–0.98) lower; patients with breast or cervical cancer had 44% (*OR*: 0.56, 95% *CI*: 0.44–0.72), 51% (*OR*: 0.49, 95% *CI*: 0.35–0.68), 46% (*OR*: 0.54, 95% *CI*: 0.40–0.74), and 36% (*OR*: 0.64, 95% *CI*: 0.49–0.83) lower odds of being satisfied with the administrative process, medical care, symptom management and overall satisfaction aspects. Compared with those with cancer stages 0–2, patients who did not know their cancer stage had relatively lower odds of being satisfied with the four aspects, except for the medical care aspect. When representatives completed the questionnaire, satisfaction rates with the administrative process, hospital environment, and symptom management were ~18–24% lower than when patients completed the survey (Table 3).

### Effect of case-mix adjustment

Hospital performance altered after case-mix adjustment. Hospital ranking based on patient satisfaction with various aspects (within a range of 5) changed (Supplementary Figure 1). Hospital performance based on patient satisfaction (better, average, or worse than average) was slightly changed in the overall satisfaction aspect and symptom management aspect after risk adjustment (Supplementary Table 2).

### Inter-hospital variation of patient satisfaction

**Figure 2:**
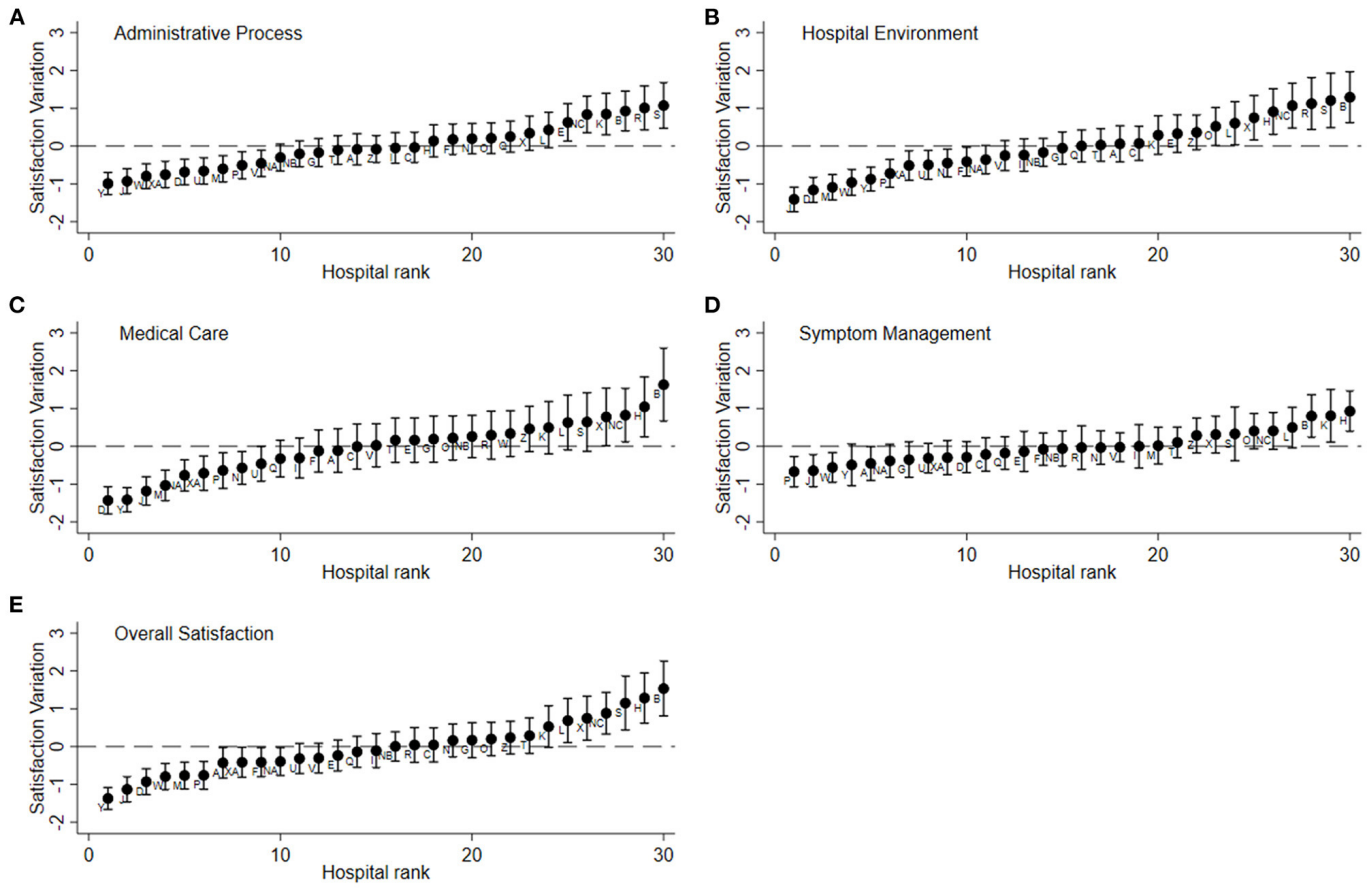
Satisfaction variation among hospitals after case-mix adjustment. **(A)** Administrative process, **(B)** hospital environment, **(C)** medical care, **(D)** symptom management, **(E)** overall satisfaction. Y axis, satisfaction variation (random effect and standard error) by hospitals; X axis, hospital rank sorted by random effect. All models are adjusted with variables: sex, age, education, annual family income, cancer type, cancer stage, self-reported health status, length of stay and actual respondent.

The caterpillar plots revealed wide patient satisfaction variation in the five aspects across hospitals (Figure 2). Even after the adjustment, significant variations in satisfaction among hospitals remained. For each aspect, high-performing hospitals with a confidence interval over 0 and low-performing hospitals with a confidence interval below 0 could be identified. The plots of the five aspects revealed that one hospitals (B) performed better in all aspects than the average, and two hospitals (J, P) performed worse in all aspects (Supplementary Table 3).

## Discussion

Patient satisfaction with various aspects in tertiary cancer hospitals in China was investigated based on a multi-center patient survey. The satisfaction level in the medical care aspect was highest, while that in the symptom management aspect was lowest. We examined patient-level characteristics associated with patient satisfaction among cancer hospitals, and using those factors as case-mix adjusters, we found there were changes in the relative level of hospital patient satisfaction after the adjustment. These findings indicate that it is necessary to standardize the patient composition in hospital evaluation researches. However, the discrepancy in hospital performance remained substantial among cancer hospitals in China after case-mix adjustment. The factors embedded in hospital structure that affect hospital performance need to be explored in the future. Overall, the methods and findings in our study were helpful for benchmarking patient satisfaction with cancer hospitals within countries and guiding hospital quality improvement efforts.

Measuring patient satisfaction with five aspects of cancer care in China provides a greater understanding of cancer care. This study presented patients' perceptions of their care including the symptom management aspect, which was rarely used in international patient experience or satisfaction surveys (14, 15, 24, 43). In this study, we found that the symptom management aspect was associated with the lowest satisfaction level (satisfaction rate of 74.56% and satisfaction score of 4.67) among Chinese cancer patients. Cancer patients worldwide may experience a high symptom burden during disease development and treatment, such as depression, pain, sleep disturbance, fatigue, and malnutrition (44, 45). As a part of palliative care, appropriate symptom management helps prolong life, enhance prognoses, and improve health-related quality of life (46, 47). More attention should be paid to symptom management aspect when conducting hospital assessment worldwide, which may promote medical education and training on this topic for medical staff and patients.

In addition, we found that patients were most satisfied with their medical care, and their satisfaction with the administrative process was comparatively lower. These findings are in accordance with previous studies for China's tertiary general hospitals, which also revealed better performance in medical care and poorer performance in process management (36). The influx of patients at tertiary hospitals in China has resulted in process management challenges (2). Within the process aspect, patients were least satisfied with waiting times, which should be addressed as a priority. Regarding the hospital environment, patients were least satisfied with the quietness and cleanliness items. The perceived need for process and environment improvement by patients alerts us to take efforts in the future.

Patient-level factors including sociodemographic characteristics (sex and age) and clinical characteristics (cancer type, cancer stage, and self-reported health status) were identified as influencing factors in the five aspects using multilevel regression models. Female and middle-aged patients were more likely to be satisfied with their service. Advanced cancer stage and worse self-reported health were risk factors associated with patient satisfaction, consistent with a previous study conducted among older cancer patients in the USA (43). The aforementioned patients are usually more depressed, worried about their disease, and concerned with care plans, leading to greater expectations and more negative perceptions. Some of patients in our study did not know their cancer stage, potentially due to undetermined staging or family members concealing the cancer stage from the patient (48). Uncertainty regarding the status of the disease may result in patient anxiety and weakens their communication flexibility with medical staff (49, 50). Actual respondent was a significant impact factor in the process, environment, and symptom management aspects. Patients who required a representative may have had more severe conditions, and the information provided by representatives reflected the collective perceptions of the patients and their representatives.

Because patient characteristics influence their perceptions of medical service, the direct comparison between hospitals with regard to patient satisfaction might be biased if there is a significant difference in patient constitution (51–53). It is suggested that when comparing satisfaction levels across hospitals, patient composition should be taken into account, and case-mix adjusted satisfaction level should be used as a benchmark (32, 54). For example, the composition of cancer types greatly differed across cancer hospitals in our study. The proportion of lung cancer patients among the sampled hospitals ranged from 6.1 to 28.7%, and that of breast cancer patients ranged from 3.8 to 39.1%. As the results of this study suggested, cancer type is a significant factor influencing patient satisfaction; thus, using it as a case-mix adjuster is essential for hospital comparison.

The patient satisfaction for each aspect was case-mix adjusted for age, sex, education, income, cancer type, cancer stage, self-reported health status, length of stay, and respondent using multilevel models. Case-mix adjustment had a modest effect on hospital comparison. First, case-mix adjustment had a modest effect on hospital rank (the rank changed within a range of 5). Second, hospital performance categories (categorized into better, average, or worse) of patient satisfaction were slightly changed in the overall satisfaction aspect and symptom management aspect after risk adjustment. Hospital performance categories were more robust compared with hospital rank before and after risk adjustment. The study on national cancer patient experience in England found 6–10 hospitals moved out of the extreme performance categories after case mix adjustment (54). The results of the national surgical quality survey in the USA showed the sufficiency of risk adjustment for accurate comparisons of hospital quality (55). While the effect of case-mix adjustment was modest for some practices in previous studies, they also found that case-mix adjustment corrected significant underestimation of scores or rates for a small proportion of practices serving vulnerable patients (56).

Wide variation in patient satisfaction among hospitals was shown after adjustment, indicating that the case-mix constitution was not decisive for difference of hospital performance in cancer care. These remaining inter-hospital variation may result from hospital features and the quality of health services provided. Therefore, for narrowing disparity of patient satisfaction among hospitals, efforts to improve the resource endowment and service quality of poor-performing hospitals should be take into consideration. The high-performing hospitals identified in analyses can act as valuable cases to offer experiences. Besides, the caterpillar plots readily revealed specific hospitals that performed worse in various aspects, which helps policymakers, researchers, and hospital managers easily identify discrepancies in hospital performance, and conduct targeted interventions for specific hospitals and corresponding aspects.

This study has several strengths. First, this was the first nationwide multi-center patient satisfaction study of cancer hospitals in China, filling a gap in the knowledge of cancer care in China. Second, this study broke down patient satisfaction into five aspects and explored substantial aspects of hospital improvement opportunities. Third, this study eliminated possible statistical inaccuracies due to the cluster effect of patients and examined the variation across hospitals using multilevel models. The inter-hospital variation was clearly visualized in the caterpillar plots. This study also has some limitations. First, this study sampled tertiary cancer hospitals in China, and the generalization of these findings was limited for other hospitals at a lower level. Second, patient information was self-reported, and recall bias may exist. However, recall bias is estimated to be low because the information collected was relatively explicit, and the recall period was short (interviews were conducted on the discharge day or the day prior). Third, our study did not include hospital characteristics and contextual factors as explanatory variables, which may reveal more sources of inter-hospital variation. Future studies should be conducted to explore the effect of health system contexts and hospital characteristics associated with patient satisfaction.

## Conclusions

This is the first nationwide multi-center patient survey regarding patient satisfaction in tertiary cancer hospitals in China. The results revealed the need to improve quality, especially in process optimization and symptom management. Substantial inter-hospital variation remained after case-mix adjustment, revealing the substantial discrepancy in patient satisfaction across hospitals and the need of efforts in quality improvement in low-performing hospitals. This study is helpful for policymakers, researchers, and hospital managers to identify problems in service quality, conduct targeted interventions, and address deficient aspects of healthcare services in cancer hospitals. Future studies could further explore factors regarding institutional features associated with patient satisfaction.

## Data availability statement

The original contributions presented in the study are included in the article/Supplementary material, further inquiries can be directed to the corresponding authors.

## Ethics statement

The studies involving human participants were reviewed and approved by the Ethics Committee of Institute of the Medical Biology of the Chinese Academy of Medical Sciences (IPB-2020-23). The patients/participants provided their written informed consent to participate in this study.

## Author contributions

ML is the guarantor. All authors contributed to the planning, conduct, analysis, and writing of this study.
