# Supplementary material for: Patient healthcare experiences of cancer hospitals in China: A multilevel modeling analysis based on a national survey
Source: Front Public Health. 2023 Feb 22;11:1059878. doi: 10.3389/fpubh.2023.1059878 (PMC9992183; doi:10.3389/fpubh.2023.1059878)
Supplement: Supplementary file 1 [file Table_1.DOCX]

**Appendix**

**eTable1 The patient satisfaction over five aspects for each hospital**

**eTable2 The change of hospital performance category over five aspects**

**eTable3 The hospital performance category after adjustment over five aspects**

**eFigure1 The change of hospital rank over five aspects**

**eTable1 The patient satisfaction on five aspects for each hospital**

| **Hospital code** | **Patient number** | **Administrative process** | | **Hospital environment** | | **Medical care** | | **Symptom management** | | **Overall satisfaction** | |
| --- | --- | --- | --- | --- | --- | --- | --- | --- | --- | --- | --- |
|  |  | **Score** | **Rate,%** | **Score** | **Rate,%** | **Score** | **Rate,%** | **Score** | **Rate,%** | **Score** | **Rate,%** |
| A | 158 | 4.75 | 84.81 | 4.69 | 89.24 | 4.79 | 93.04 | 4.65 | 66.20 | 4.75 | 82.91 |
| B | 168 | 4.87 | 94.05 | 4.84 | 97.02 | 4.99 | 100.00 | 4.88 | 91.67 | 4.96 | 98.21 |
| C | 142 | 4.69 | 80.99 | 4.60 | 85.92 | 4.85 | 92.96 | 4.55 | 67.12 | 4.80 | 85.92 |
| D | 148 | 4.58 | 67.57 | 4.40 | 62.16 | 4.68 | 72.30 | 4.60 | 65.91 | 4.61 | 67.57 |
| E | 160 | 4.78 | 91.88 | 4.71 | 90.63 | 4.77 | 93.75 | 4.62 | 72.00 | 4.74 | 83.75 |
| F | 157 | 4.72 | 84.08 | 4.59 | 79.62 | 4.84 | 92.36 | 4.66 | 72.41 | 4.75 | 80.25 |
| G | 164 | 4.68 | 80.49 | 4.61 | 85.98 | 4.87 | 94.51 | 4.55 | 67.69 | 4.83 | 89.02 |
| H | 149 | 4.56 | 85.23 | 4.77 | 94.63 | 4.96 | 97.99 | 4.91 | 92.22 | 4.91 | 96.64 |
| I | 158 | 4.75 | 84.18 | 4.74 | 85.44 | 4.88 | 91.14 | 4.72 | 77.27 | 4.83 | 87.34 |
| J | 149 | 4.47 | 64.43 | 4.31 | 57.72 | 4.64 | 75.84 | 4.47 | 55.41 | 4.55 | 63.09 |
| K | 153 | 4.82 | 94.12 | 4.75 | 90.85 | 4.86 | 96.08 | 4.96 | 97.5 | 4.86 | 92.81 |
| L | 149 | 4.79 | 89.26 | 4.77 | 93.29 | 4.91 | 96.64 | 4.86 | 88.31 | 4.89 | 93.96 |
| M | 149 | 4.58 | 71.14 | 4.50 | 65.77 | 4.66 | 80.54 | 4.68 | 77.14 | 4.63 | 72.48 |
| N | 168 | 4.71 | 85.12 | 4.60 | 79.17 | 4.72 | 86.31 | 4.63 | 72.84 | 4.76 | 87.50 |
| O | 175 | 4.76 | 86.29 | 4.74 | 92.00 | 4.9 | 94.29 | 4.80 | 84.04 | 4.83 | 88.57 |
| P | 149 | 4.56 | 74.50 | 4.46 | 75.17 | 4.75 | 87.25 | 4.51 | 60.23 | 4.69 | 75.17 |
| Q | 175 | 4.69 | 86.86 | 4.61 | 86.86 | 4.81 | 90.29 | 4.68 | 72.41 | 4.77 | 85.71 |
| R | 151 | 4.87 | 95.36 | 4.73 | 96.69 | 4.85 | 94.7 | 4.67 | 77.27 | 4.76 | 87.42 |
| S | 144 | 4.88 | 95.83 | 4.83 | 97.22 | 4.86 | 97.22 | 4.74 | 87.5 | 4.91 | 97.22 |
| T | 168 | 4.68 | 82.14 | 4.66 | 86.90 | 4.87 | 94.05 | 4.77 | 78.45 | 4.86 | 89.88 |
| U | 145 | 4.48 | 69.66 | 4.56 | 77.24 | 4.79 | 86.21 | 4.61 | 67.33 | 4.71 | 80.00 |
| V | 162 | 4.56 | 75.31 | 4.62 | 82.72 | 4.83 | 93.21 | 4.69 | 76.19 | 4.71 | 82.10 |
| W | 161 | 4.50 | 68.32 | 4.31 | 71.43 | 4.78 | 94.41 | 4.54 | 61.29 | 4.63 | 73.29 |
| X | 146 | 4.73 | 87.67 | 4.73 | 93.84 | 4.91 | 97.26 | 4.76 | 82.72 | 4.87 | 93.84 |
| Y | 192 | 4.43 | 64.58 | 4.47 | 72.40 | 4.5 | 73.96 | 4.50 | 62.5 | 4.48 | 61.46 |
| Z | 186 | 4.66 | 81.18 | 4.71 | 90.32 | 4.85 | 95.16 | 4.67 | 80.23 | 4.80 | 88.71 |
| XA | 148 | 4.41 | 70.27 | 4.42 | 79.05 | 4.77 | 85.81 | 4.57 | 68.06 | 4.73 | 80.41 |
| NA | 171 | 4.61 | 78.95 | 4.62 | 81.29 | 4.78 | 84.80 | 4.54 | 64.86 | 4.77 | 80.12 |
| NB | 197 | 4.69 | 80.71 | 4.67 | 83.76 | 4.83 | 94.42 | 4.62 | 74.67 | 4.80 | 85.79 |
| NC | 205 | 4.85 | 93.66 | 4.86 | 96.10 | 4.94 | 97.56 | 4.82 | 86.02 | 4.91 | 95.12 |

**eTable2** **The change of hospital performance category over five aspects**

1. **Process management**

| Before case-mix adjustment | After case-mix adjustment | | |
| --- | --- | --- | --- |
|  | Better | Average | Worse |
| Better | 6 | 0 | 0 |
| Average | 0 | 15 | 0 |
| Worse | 0 | 0 | 9 |

1. **Hospital environment**

| Before case-mix adjustment | After case-mix adjustment | | |
| --- | --- | --- | --- |
|  | Better | Average | Worse |
| Better | 8 | 0 | 0 |
| Average | 0 | 12 | 0 |
| Worse | 0 | 0 | 10 |

1. **Medical care**

| Before case-mix adjustment | After case-mix adjustment | | |
| --- | --- | --- | --- |
|  | Better | Average | Worse |
| Better | 4 | 0 | 0 |
| Average | 0 | 17 | 0 |
| Worse | 0 | 0 | 9 |

1. **Symptom management**

| Before case-mix adjustment | After case-mix adjustment | | | |
| --- | --- | --- | --- | --- |
|  | Better | Average | | Worse |
| Better | 3 | | 2 | 0 |
| Average | 0 | | 22 | 0 |
| Worse | 0 | | 0 | 3 |

1. **Overall satisfaction**

| Before case-mix adjustment | After case-mix adjustment | | |
| --- | --- | --- | --- |
|  | Better | Average | Worse |
| Better | 6 | 1 | 0 |
| Average | 0 | 12 | 1 |
| Worse | 0 | 1 | 9 |

Note: Better means confidence interval of satisfaction variation is over 0; average means confidence interval of satisfaction variation contains 0; worse means confidence interval of satisfaction variation is below 0.

**eTable3 The hospital performance category after adjustment over five aspects**

| **Hospital code** | **Administrative process** | **Hospital environment** | **Medical care** | **Symptom management** | **Overall satisfaction** |
| --- | --- | --- | --- | --- | --- |
|  |  |  |  |  |  |
| A | average | average | average | average | worse |
| B | better | better | better | better | better |
| C | average | average | average | average | average |
| D | worse | worse | worse | average | worse |
| E | better | average | average | average | average |
| F | average | worse | average | average | worse |
| G | average | average | average | average | average |
| H | average | better | better | better | better |
| I | average | average | average | average | average |
| J | worse | worse | worse | worse | worse |
| K | better | average | average | better | average |
| L | average | better | average | average | better |
| M | worse | worse | worse | average | worse |
| N | average | worse | worse | average | average |
| O | average | better | average | average | average |
| P | worse | worse | worse | worse | worse |
| Q | average | average | average | average | average |
| R | better | better | average | average | average |
| S | better | better | average | average | better |
| T | average | average | average | average | average |
| U | worse | worse | worse | average | average |
| V | worse | average | average | average | average |
| W | worse | worse | average | worse | worse |
| X | average | better | better | average | better |
| Y | worse | worse | worse | average | worse |
| Z | average | average | average | average | average |
| XA | worse | worse | worse | average | worse |
| NA | average | average | worse | average | worse |
| NB | average | average | average | average | average |
| NC | better | better | better | average | better |

Note: Better means confidence interval of satisfaction variation is over 0; average means confidence interval of satisfaction variation contains 0; worse means confidence interval of satisfaction variation is below 0.

**eFigure1 The change of hospital rank over five aspects**


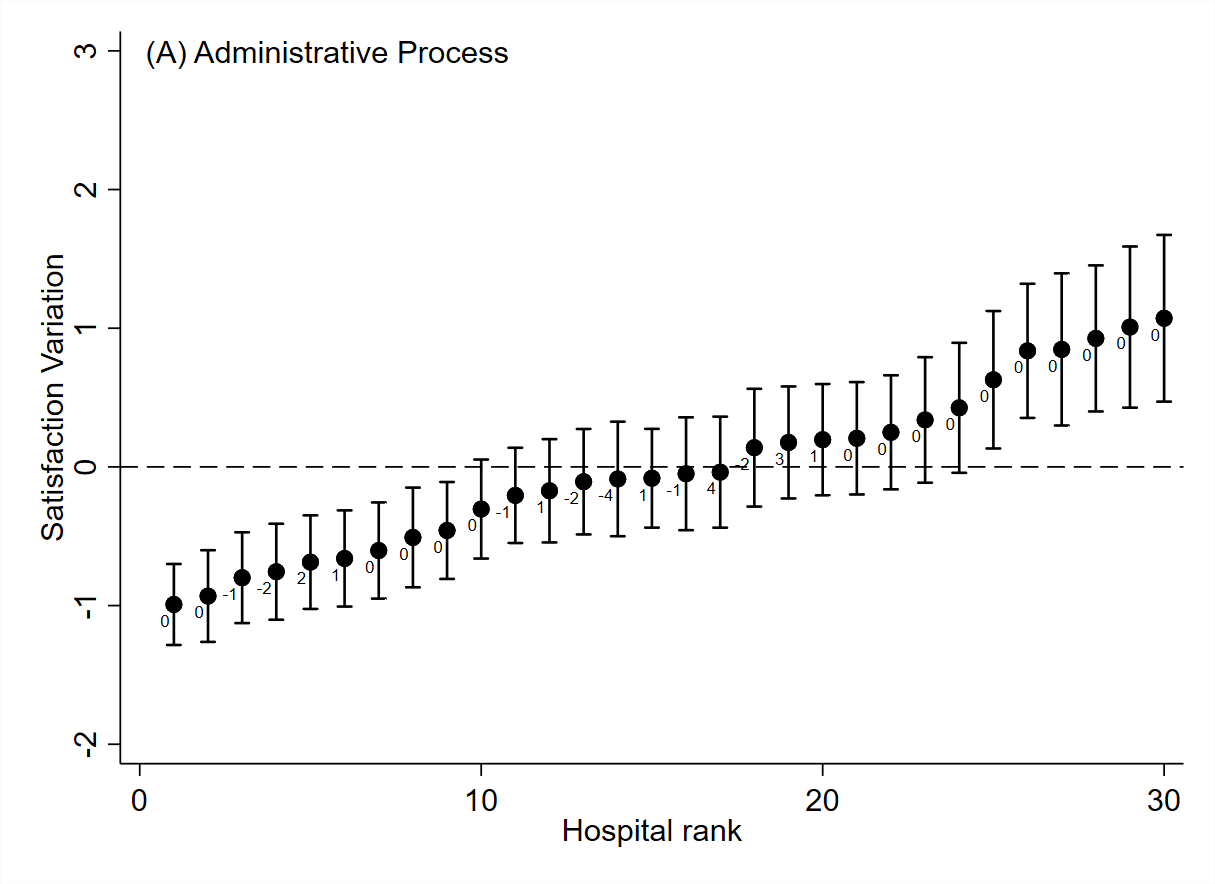


Note: The value label of pot is the hospital rank change after adjustment. The change range of hospital rank is [-4,4].


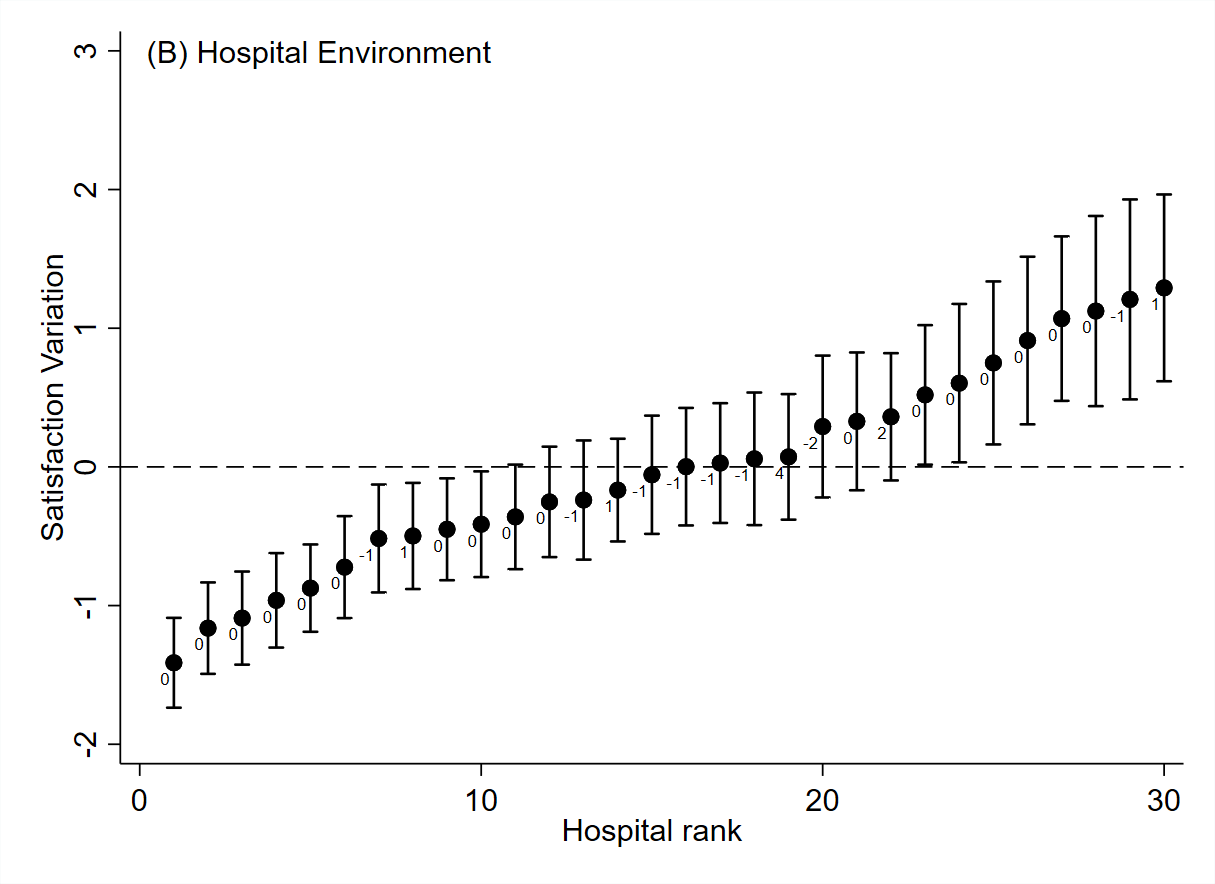


Note: The value label of pot is the hospital rank change after adjustment. The change range of hospital rank is[-2,4]


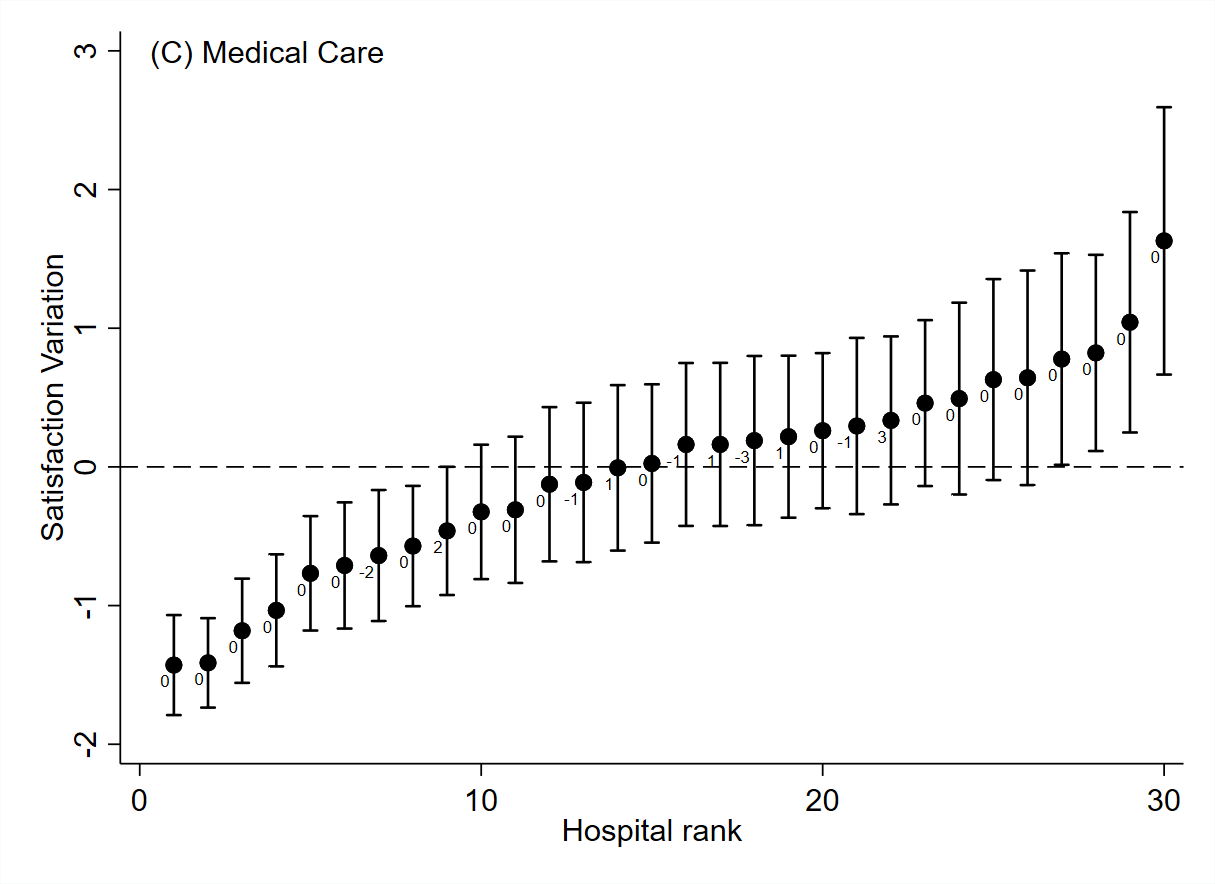


Note: The value label of pot is the hospital rank change after adjustment. The change range of hospital rank is [-3,3]


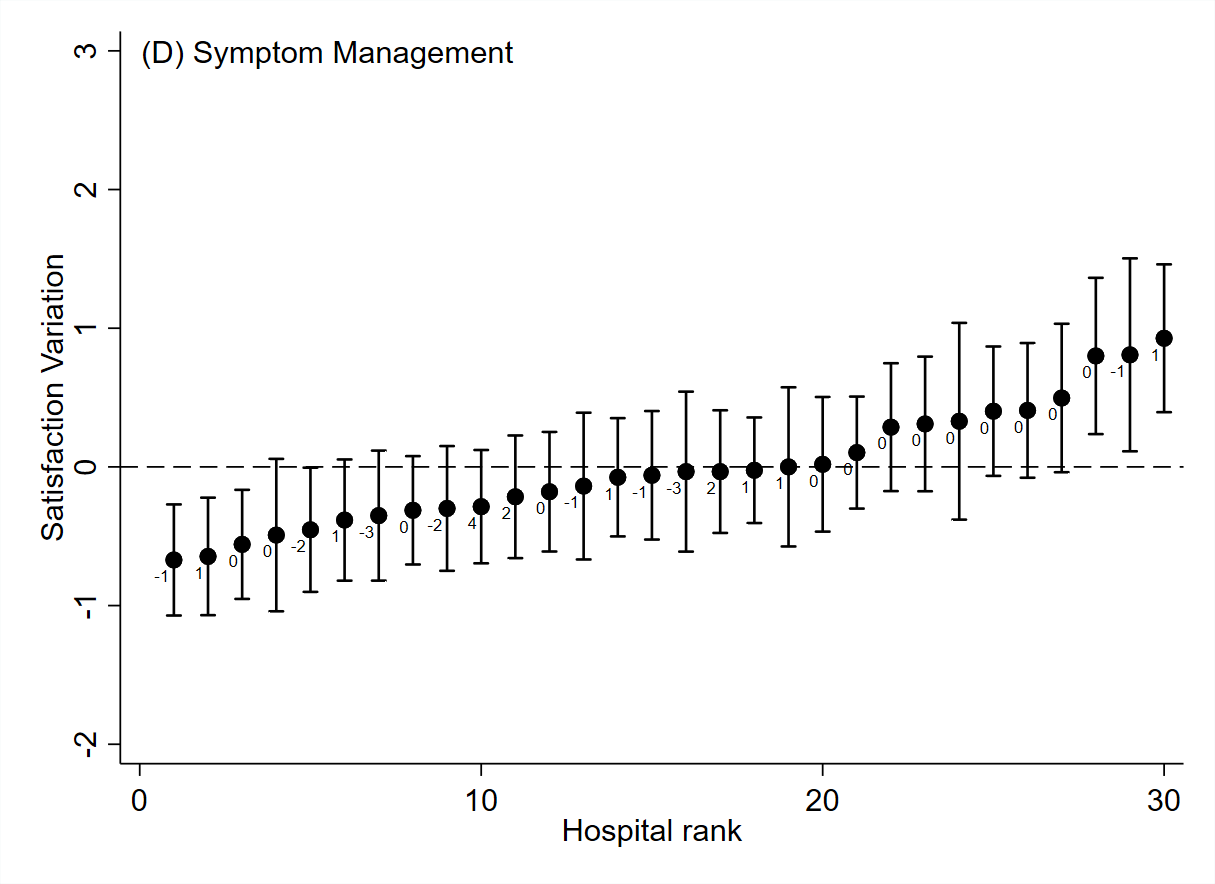


Note: The value label of pot is the hospital rank change after adjustment. The change range of hospital rank is [-3,4].


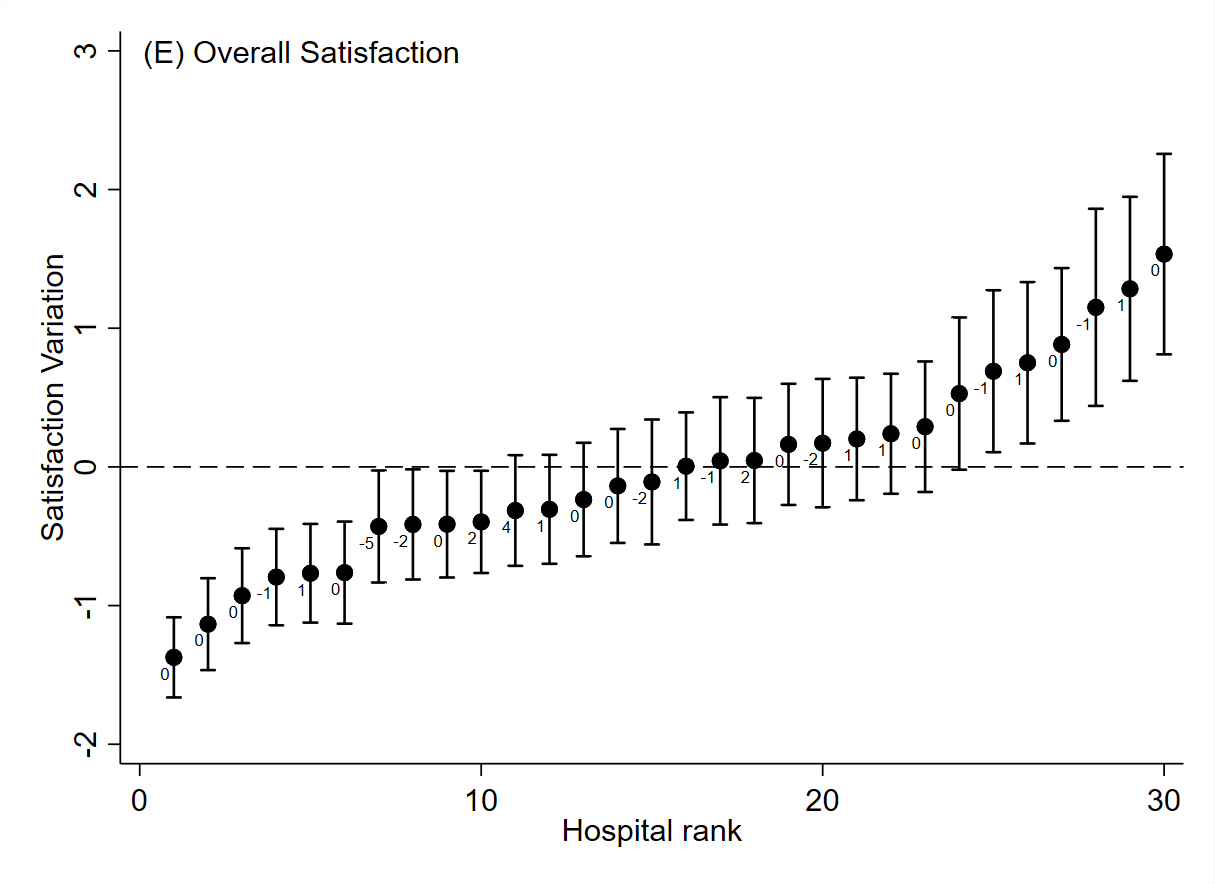


Note: The value label of pot is the hospital rank change after adjustment. The change range of hospital rank is [-5,4].
